# A study on neurodevelopmental outcomes in infants with congenital hypothyroidism highlights the importance of periodic developmental assessment

**DOI:** 10.1016/j.jped.2025.01.001

**Published:** 2025-02-06

**Authors:** A.S. van Trotsenburg, N. Zwaveling-Soonawala

**Affiliations:** a University of Amsterdam, Emma Children's Hospital Amsterdam University Medical Center, Department of Pediatric Endocrinology, Amsterdam, The Netherlands; b Amsterdam Gastroenterology Endocrinology Metabolism, Amsterdam, The Netherlands; c European Reference Network on Rare Endocrine Conditions (Endo-ERN), Amsterdam, The Netherlands

In this issue of the Jornal the Pediatria, Romero et al.^1^ report the results of a study on neurodevelopmental assessment in infants with congenital hypothyroidism (CH) detected by the Brazilian newborn screening program for CH.

Congenital hypothyroidism can be divided into primary CH, of thyroidal origin, or central CH, of hypothalamic-pituitary origin. The incidence of primary CH is estimated at 1 in 2000–4000 newborns while central CH is rarer, with the highest reported incidence of 1 in 13,000–16:000.^2^, ^3^, ^4^ Most cases of primary CH are due to abnormal formation of the thyroid gland, so-called thyroid dysgenesis accounting for 60–70% of cases. Thyroid dyshormonogenesis accounts for the remaining 30–40% of primary CH cases and is caused by defective thyroid hormone (TH) synthesis by a structurally normal thyroid.^5^ Central CH may be part of combined pituitary hormone deficiencies (CPHD, 60–70% of cases), or may occur as isolated TSH deficiency (30–40% of cases).^4^

Thyroid hormone is indispensable for normal pre- and postnatal brain development, and when left untreated CH causes irreversible brain damage. Since signs and symptoms in the neonatal period are typically subtle or even absent due to the protective effects of maternal TH crossing the placenta, the diagnosis of CH in the neonatal period is often missed. In 1974, NBS for CH was started as a pilot in the province of Quebec, Canada, aiming at early detection and treatment in order to prevent irreversible neurodevelopmental delay.^6^ This pilot and similar initiatives in North America were found to be very successful, not only with respect to early detection but also in the prevention of intellectual disability due to primary CH.^7^ This led to the implementation of NBS programs for CH in other regions of Canada and many other countries worldwide.^8^ The Canadian pilot started with the measurement of total thyroxine (T4) in filter paper blood spots obtained after the first days of life. To speed up the detection of CH, shortly thereafter TSH measurement was added in newborns with the lowest T4 concentrations. With this, the first T4-reflex-TSH-based program was developed. New programs adopted this approach or chose for simultaneous measurement of T4 and TSH ("T4+TSH-based"), or measurement of TSH only. Currently, 101 of the 193 United Nations countries have a documented national NBS for the CH program. The vast majority of countries employ a TSH-based approach while only seven (states or regions within) countries use a T4+TSH- or T4-reflexTSH-based approach.^9^

While TSH-based NBS programs effectively detect primary CH they unfortunately miss cases of central CH. The advantage of a combined T4 and TSH approach is the ability to detect both primary and central CH.

Since the implementation of NBS for CH various studies on neurodevelopment in early detected and treated CH have been performed.^2^^,^^3^ Newborn screening has proven to be very efficient in preventing severe developmental delay in infants with CH. Based on the results of these mainly retrospective studies NBS programs were optimized (e.g., blood collection for T4 or TSH measurement earlier after birth, and better and faster measurement techniques to shorten the time between birth and the first NBS result), and TH treatment was improved (e.g., increasing the levothyroxine [LT4] starting dose from less than 10 micrograms per kg to 10–15 microgram per kg per day. With this, nowadays most early detected and adequately treated children with CH have or should have a normal neurodevelopmental and school outcome. Intellectual disability - defined as an IQ < 70 - has virtually become a condition in the past.

In this context, “adequately treated” means oral LT4 (in the form of tablets, crushed and administered with a little water to neonates and infants), started before the age of 14 days at a dosage of 10–15 microgram per day, with normalization of serum FT4 and TSH within the following 2 wk (= approximately within the neonatal period). LT4 dosing should be guided by frequent laboratory measurements to maintain TSH and FT4 within, and in the upper half of the age-specific reference interval, respectively, and to avoid LT4 under- or overtreatment. The first clinical and biochemical follow-up consultation/evaluation should take place one to 2 wk after the start of LT4 (to check for medication adherence and to adjust the LT4 dose in case of clinical or biochemical overtreatment). Subsequent consultations should be scheduled every 2 wk until normalization of serum TSH. Follow-up consultations should also be used to inform parents about their child's condition and to emphasize the importance of daily LT4 administration. Thereafter, the evaluation frequency can be lowered to once every one to three months until the age of 12 months, every 2 to four months until the age of three years, followed by evaluations every 3 to 6 months until growth is completed.^2^^,^^3^

Despite the favorable results from neurodevelopmental outcome studies, especially children with severe CH - defined as a serum FT4 < 5 pmol/L - may have subtle cognitive (e.g., memory problems) and motor deficits (clumsiness) that may result from TH deficiency in utero, not completely reversed by LT4 treatment started shortly after birth.^2^ In addition, children with CH have a higher risk of hearing loss - also related to CH severity - that can impair speech development. Finally, some older children with CH have behavioral problems/attention deficit which has been suggested to be related to periods of TH overtreatment early in life.^2^^,^^3^

With this in mind, both the European Society for Pediatric Endocrinology (ESPE) and the European Society for Endocrinology (ESE), as well as the American Academy of Pediatrics (APA) advise periodical evaluation of developmental and school progress.^2^^,^^3^ Given the higher risk of hearing loss, all neonates with CH should undergo hearing testing. This should also be done when there is clinical concern about a hearing deficit or abnormal language development. Behavior or attention problems may be reason for additional evaluation, for instance by a child psychologist or psychiatrist. In the small proportion of children with CH that display significant neurodevelopment delay, other potential causes should be investigated, like syndromic forms of CH with brain abnormalities or other conditions impairing brain development (if necessary, in collaboration with a pediatric neurologist or clinical geneticist), (a history of) poor adherence to treatment, or (lower) socioeconomic status of the family.^2^^,^^3^

In this issue of the Jornal the Pediatria, Romero et al.^1^ report developmental assessment results of 77 Brazilian infants with CH detected by NBS and treated with TH (CH group). Forty-nine disease-free infants attending daycare served as healthy controls.

The two groups were similar with respect to gender distribution, gestational age, birth weight, and age at developmental assessment (12 ± 6.4 and 13 ± 4.6 months in the CH group and controls, respectively). Infants with health conditions other than CH that could affect child development were excluded from participation.

The three main research questions were: 1) are infants with CH at greater risk for developmental delay compared to controls, 2) are infants with CH treated within or after the neonatal period at greater risk for developmental delay compared to controls, and 3) are infants with diagnostic/confirmatory TSH levels up to or above 30 mIU/l at greater risk for developmental delay compared to controls.

In 46 of the 77 infants with CH, TH treatment was started within the neonatal period (defined as age ≤ 28 days), in the other 31 treatment was started after the neonatal period. Twenty-six infants with CH had a pre-treatment diagnostic TSH level > 30 mIU/L - infants with more severe CH -, in 51 it was ≤ 30 mIU/L. Twenty-one of the 46 infants (41.7%) in whom TH treatment was started within the neonatal period had a diagnostic TSH > 30 mIU/L, while only 5 of the 31 infants (16.1%) who were started on TH treatment after the neonatal period had a TSH > 30 mIU/L.

Cognitive, receptive and expressive language, and fine and gross motor development were assessed by the Bayley Scales of Infant and Toddler Development Screening Test, Third Edition. The experienced examiners were blind to neonatal, NBS, and TH treatment data. Test scores on these domains were dichotomized: a domain score ≥ 25th percentile (or ≥ −0.67 SD) was noted as “lower risk”, a score < 25th percentile (or < −0.67 SD) as “greater risk” for developmental delay.

In all three comparisons, the CH group performed worse than controls on domains of receptive language, fine motor and gross motor. Differences were greatest for infants with CH and a diagnostic TSH > 30 mIU/L. With respect to the domain cognition, there were no differences between the total CH group and controls, and between the CH subgroups “TH started after the neonatal period” and “diagnostic TSH ≤ 30 mIU/L”, and controls. However, compared to controls, the CH subgroups “TH started within the neonatal period” and “diagnostic TSH > 30 mIU/L” performed considerably worse, with a larger percentage of infants in the "greater risk" group.

The authors conclude that their “study demonstrates a greater risk for development delay in cognition, receptive language, and both fine and gross motor skills in infants with CH, especially in those with serum TSH levels in the confirmatory test exceeding 30 mUI/L”, underscoring “the need for increased attention and monitoring of infant development for these infants.” With this, the authors advocate for the inclusion of neurodevelopmental monitoring in the Brazilian neonatal screening reference services (SRTN). Not only for early detection of delays but - if indicated - also for intervention, like speech therapy, physiotherapy, or support by a child psychologist.^1^, ^2^, ^3^

Romero et al.^1^ are to be praised for conducting this study and reporting their results. What they are advocating is in line with the recommendations of the ESPE ESE, and the APA.

Despite the favorable results from various neurodevelopmental outcome studies, subtle cognitive impairment and motor deficits are reported in children with CH despite early treatment. This seems mainly the case in the subset with severe CH - defined as a confirmatory serum FT4 < 5 pmol/L.^2^^,^^3^ This may result from TH deficiency in utero which is not completely reversed by LT4 treatment started shortly after birth, within the first 2 wk.

In this study from Brazil, early treatment is defined as treatment initiated within the neonatal period (the first 28 days of life). It would be interesting to know whether infants treated within the first 14 days of life show better neurodevelopment. Of course, the authors acknowledge that such a calculation may not be possible due to the relatively small numbers. In addition, data on TH treatment are lacking, and in particular, whether LT4 treatment was carried out in accordance with the latest recommendations (see above). If these data can be retrieved, authors together with the SRTN may have the opportunity to optimize their NBS for the CH program and, if necessary, treatment practice.

Finally, a point of attention. When performing developmental outcome studies, it is important to have a comparable control group. While the authors included healthy infants and excluded diseases that may affect development, a remarkably high percentage of controls received the classification "lower risk" in the domains “receptive language, fine and gross motor skills, and cognition” (95.5%, 91.8%, 91.8% and 93.9%, respectively), following from a test score ≥ 25th percentile according to the standardization of the Bayley-III Screening Test. Because intelligence is highly heritable,^10^ it cannot be ruled out that these percentages that are considerably higher than the expected 75% are due to differences in socioeconomic class between the controls and the population on which the Bayley-III Screening Test was standardized (with an on average higher socioeconomic class of the parents of controls).

The year 2024 marks the 50th anniversary of the introduction of NBS for CH. Newborn screening has proven to be very effective in facilitating early detection and treatment of CH, thereby preventing severe developmental delay. However neurodevelopmental deficits are still reported in infants with early detected CH, mostly in the most severe cases. The study by Romero et al. in this issue of the Jornal the Pediatria, highlights the importance of periodical evaluation of developmental and school progress in children with CH.

## Conflicts of interest

The authors declare no conflicts of interest.
